# Laboratory Verification of a *BRCA1* and *BRCA2* Massively Parallel Sequencing Assay from Wet Bench to Bioinformatics for Germline DNA Analysis

**DOI:** 10.1055/s-0041-1726338

**Published:** 2021-03-16

**Authors:** Kok-Siong Poon, Lily Chiu, Karen Mei-Ling Tan

**Affiliations:** 1 Department of Laboratory Medicine, National University Hospital Singapore, Singapore

**Keywords:** *BRCA1*, *BRCA2*, massively parallel sequencing, NGS, Multiplicom

## Abstract

**Introduction** A robust genetic test for *BRCA1* and *BRCA2* genes is necessary for the diagnosis, prognosis, and treatment of patients with hereditary breast and ovarian cancer. We evaluated a commercial amplicon-based massively parallel sequencing (MPS) assay, BRCA MASTR Plus on the MiSeq platform, for germline *BRCA* genetic testing.

**Methods** This study was performed on 31 DNA from cell lines and proficiency testing samples to establish the accuracy of the assay. A reference cell line DNA, NA12878 was used to determine the reproducibility of the assay. Discordant MPS result was resolved orthogonally by the current gold-standard Sanger sequencing method.

**Results** The analytical accuracy, sensitivity, and specificity for variant detection were 93.55, 92.86, and 100.00%, respectively. Both sequencing depth and variant allele frequencies were highly reproducible by comparing the NA12878 DNA tested in three separate runs. The single discordant result, later confirmed by Sanger sequencing was due to the inability of the MASTR Reporter software to identify a 40-bp deletion in *BRCA1* .

**Conclusion** The BRCA MASTR Plus assay on the MiSeq platform is accurate and reproducible for germline *BRCA* genetic testing, making it suitable for use in a clinical diagnostic laboratory. However, Sanger sequencing may still serve as a confirmatory method to improve diagnostic capability of the MPS assay.

## Introduction

Hereditary breast and ovarian cancer (HBOC) is an autosomal dominant cancer syndrome frequently caused by germline pathogenic variants in the two DNA repair genes, *BRCA1* and *BRCA2* . Due to high penetrance, approximately 50% of women with *BRCA* pathogenic variants will be diagnosed with breast cancer by age of 70 years. 1 For ovarian cancer, these estimates were 40 and 18% of women with mutant *BRCA1* and *BRCA2* genes, respectively. 1 Several poly ADP ribose polymerases (PARP) inhibitors have been approved for therapy in patients with HBOC syndrome with germline *BRCA* pathogenic variants. 2 In patients with >30% variant allele frequency (VAF) of pathogenic *BRCA* variants from tumor profiling, genetic testing of germline variant is recommended. 3 Genetic testing of the *BRCA* genes plays a vital role to allow identification of carriers of pathogenic variants and increased screening for early detection of breast and ovarian cancers in these individuals. A robust laboratory assay is crucial to enable genetic testing of *BRCA1* and *BRCA2* genes on which risk assessment, patient management, and therapeutic assignment in HBOC patients rely.

The *BRCA1* (OMIM: 113705) and *BRCA2* (OMIM: 600185) genes, located at 17q21.31 and 13q13.1, are large genes with 24 and 27 exons encoding 1,863 and 3,418 amino acids, respectively. They are tumor-suppressor genes in which loss-of-function variants are associated with increased risk of HBOC syndrome. A wide spectrum of pathogenic variants is detectable throughout the coding and splice site regions of the *BRCA* genes. These genetic alterations are heterogeneous, including single nucleotide variants (SNVs), small insertions and deletions (indels) affecting a short stretch of nucleotides, large indels at exonic level and copy number variants (CNV). With the advent of massively parallel sequencing (MPS), the laboratory accessibility to sequencing the two large *BRCA* genes is improved. A recent international survey 4 revealed that 93% of the laboratories utilize MPS platforms for sequencing the *BRCA* genes. In this study, we evaluated a commercial amplicon-based MPS assay, BRCA MASTR Plus (Multiplicom, Niel, Belgium) on the MiSeq platform (Illumina; San Diego, California, United States), for germline *BRCA1* and *BRCA2* genetic testing.

## Methods

### DNA Samples

Samples tested in this study were cell line DNA from Coriell Institute for Medical Research ( *n* = 10), namely, NA13714, NA14091, NA14624, NA14639, NA14788, NA14805, NA14623, NA14622, NA14170, and NA12878. Notably, NA12878 is a reference cell line characterized by the Genome in a Bottle (GIAB) Consortium hosted by National Institute of Standards and Technology (NIST). 5 Twenty-one DNA samples accrued from College of American Pathologists/American College of Medical Genetics (CAP/ACMG) *BRCA* 1/2 Sequencing External Quality Assurance (EQA) Program were also tested in this study.

### BRCA MASTR Plus Assay Library Preparation and Sequencing

Concentrations of DNA samples were measured using NanoDrop 2000 (Thermo Fisher Scientific, Waltham, Massachusetts, United States). Working DNA samples were diluted with nuclease-free water (Invitrogen, Waltham, Massachusetts, United States) to a concentration of 10 ng/µL. Five µL of diluted DNA was subject to four multiplex polymerase chain reactions (PCRs) with reagents supplied in BRCA MASTR Plus kit (Multiplicom) according to manufacturer's instructions. Five µL of PCR products from the multiplex PCR reactions were subject to electrophoresis using 2% (w/v) agarose gel in Tris-Borate-EDTA (TBE) buffer at 100 V for 30 minutes to verify the presence of PCR products with sizes exceeding 150 base pairs. Equal volumes of multiplex PCR products were pooled and purified with AMPure XP beads (Agencourt; Beverly, Massachusetts, United States). Universal PCR was performed on purified pooled PCR products with MID p7 and p5 adaptor and primer mixes supplied in drMID for Illumina NGS systems kit (Multiplicom). Five µL of the universal PCR products were subject to electrophoresis using 2% (w/v) agarose gel in TBE buffer at 100 V for 30 minutes to verify the presence of PCR products with sizes exceeding 200 base pairs. The universal PCR products purified with AMPure XP beads were diluted to 4 nM in TE buffer (Thermo Fisher Scientific) and pooled into a single library. The library was diluted to 12pM and denatured using 0.2 N sodium hydroxide (NaOH). MPS was performed on the MiSeq system (Illumina) using MiSeq Reagent Micro Kit v2 (300 cycles).

### Bioinformatics

The demultiplexed FASTQ sequence files were uploaded to MASTR Reporter v1.2.1, proprietary web-based software by Multiplicom. The application of BRCA MASTR Plus Dx Germline was selected to analyze the sequencing data in this study. For variant analysis, the minimum coverage depth and allele frequency were specified at 40× and 20%, respectively. Variants were classified according to the 2015 ACMG/AMP guidelines. 6

### Sanger Confirmation

A set of primers was designed using Primer3 software to amplify exon 11 of the *BRCA1* gene with expected PCR product size of 430 base pairs (bp). The forward and reverse primers were 5′cagaaactgccatgctcaga3′ and 5′tgaggggacgctcttgtatt3′, respectively. PCR was performed using HotStarTaq Plus Master Mix Kit (QIAGEN; Hilden, Germany) on 50 ng of DNA input. PCR products were purified with GeneAll Expin Kit (GeneAll Biotechnology, Seoul, Korea) and subject to cycle sequencing reaction using the same forward and reverse PCR primers with BigDye Terminator v3.1 Cycle Sequencing kit (Thermo Fisher Scientific). Sanger sequencing was performed on cycle sequencing products purified with DyeEx 2.0 Spin kit (QIAGEN) on ABI 3130 Genetic Analyzer (Thermo Fisher Scientific). Sequence analysis was performed using ATF software (Conexio Genomics, Fremantle, Australia).

### Statistical Analysis

Accuracy was calculated as the number of true positives and true negatives divided by the sum of true positives, true negatives, false positives, and false negatives using an online statistical software, MEDCALC.

## Results

### Sequencing Depth and Reproducibility of the Assay

**Fig. 1:**
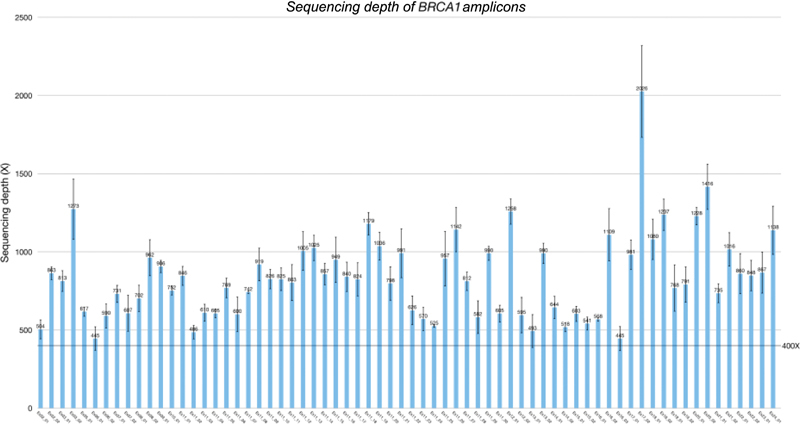
Sequencing depth of *BRCA1* amplicons. Bars represent mean and error bars represent standard deviation of three separate runs.

**Fig. 2:**
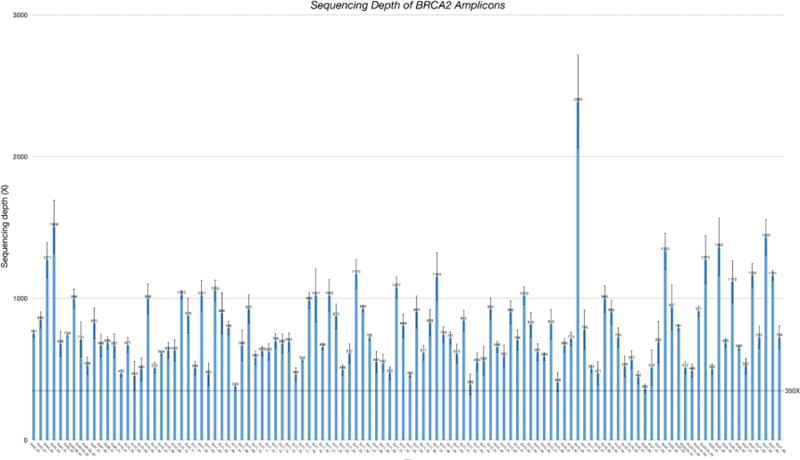
Sequencing depth of *BRCA2* amplicons. Bars represent mean and error bars represent standard deviation of three separate runs.

**Fig. 3:**
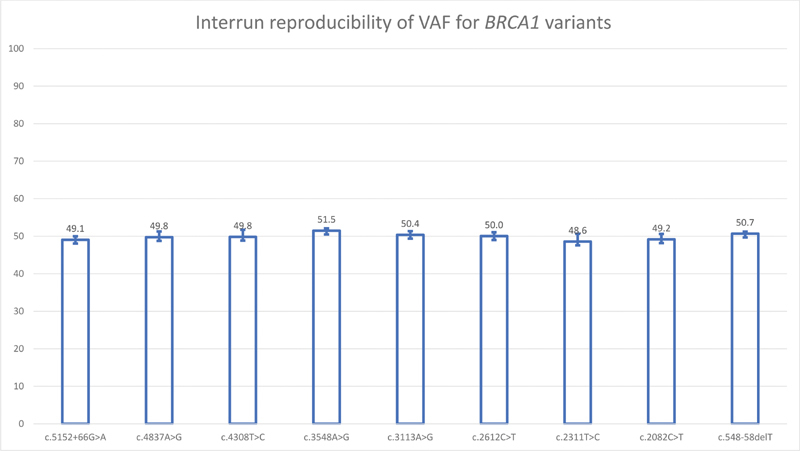
Interrun reproducibility of variant allele frequency for *BRCA1* variants. Bars represent mean and error bars represent standard deviation of three separate runs.

**Fig. 4:**
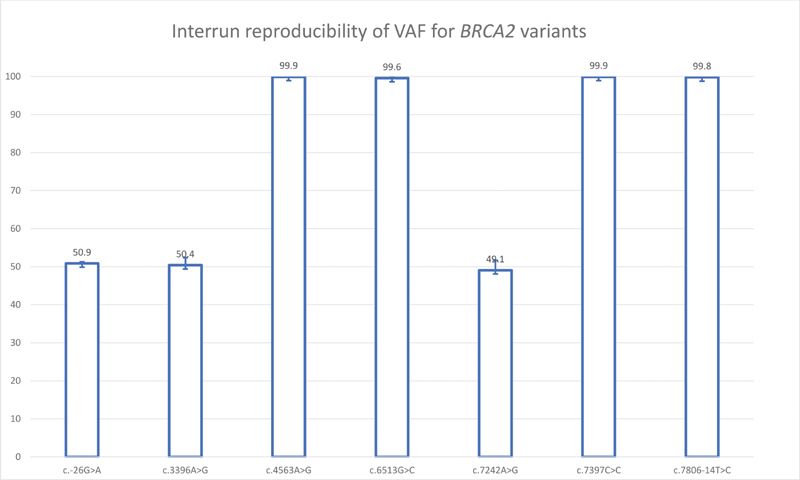
Interrun reproducibility of variant allele frequency for *BRCA2* variants. Bars represent mean and error bars represent standard deviation of three separate runs.

With a pooling strategy with 10 samples per library, the minimum sequencing depth was above 400× and 350× for *BRCA1* (69 amplicons) and *BRCA2* (112 amplicons), respectively ( Figs. 1 and 2 ). Comparing three batches of sequencing runs, the average sequencing depths per sample were 957× (66; mean [standard deviation (SD)]) and 873× (63) for the *BRCA1* and *BRCA2* , genes respectively. DNA from NA12878 was tested in a pooled library in three separate runs. Nine heterozygous *BRCA1* variants and three heterozygous and four homozygous *BRCA2* variants were consistently identified with highly reproducible VAF ( Figs. 3 and 4 ). All *BRCA* variants identified by the current assay were in concordance with the variant datasets available from the Genetic Testing Reference Material (GeT-RM) browser at the National Center for Biotechnology Information (NCBI).

### Analytical Validity of the Assay

**Fig. 5:**
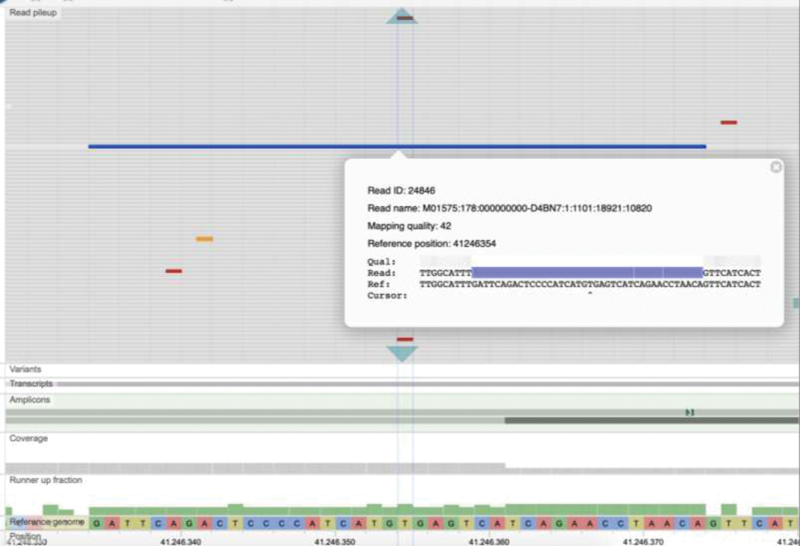
A long event with 40-bp deletion was equivocally detected on one of the *BRCA1* exon 11 amplicons of the BRCA MASTR Plus MPS assay. Mapping of reads to the *BRCA1* genomic region was visualized by read pileup in MASTR Reporter. The read with deleted region was abbreviated in blue color. The details including read ID, read name, mapping quality, reference position and nucleotide sequences were shown in the browser. The deletion was later confirmed by PCR and Sanger sequencing (illustrated in Fig. 6 ) to be c.1175_1214del40, which was the expected variant according to CAP. CAP, College of American Pathologists; MPS, massively parallel sequencing; PCR, polymerase chain reaction.

**Fig. 6:**
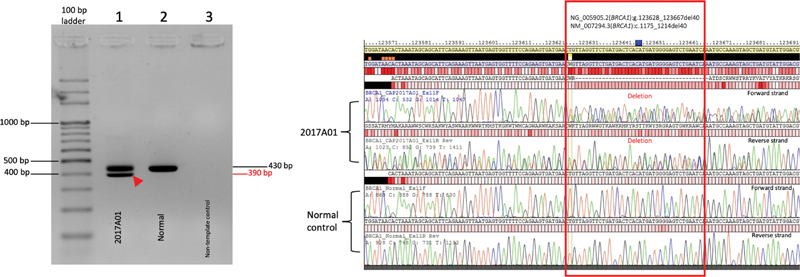
Confirmation of an equivocal long event detected by MASTR Reporter software using PCR–Sanger sequencing. The *BRCA1* genomic region of interest was PCR amplified (left) and followed by Sanger sequencing of the PCR products. The deletion event, c.1175_1214del40 was verified by alignment to the *BRCA1* genomic RefSeq NG_005905.2 (right). PCR, polymerase chain reaction.

In addition to NA12878, DNA samples from nine cell lines with known *BRCA* variants were evaluated ( Table 1 ). All results were concordant with the data on these cell lines available from the Coriell Institute for Medical Research. Twenty-one blinded DNA samples, accrued from the CAP/ACMG BRCA1/2 sequencing program, were tested ( Table 1 ). From these samples, eight heterozygous frameshift variants, three heterozygous stop-gain variants, and three heterozygous SNVs were correctly identified in the *BRCA* genes. Notably, a 40-bp deletion variant in the *BRCA1* gene was not conclusively reported by the MASTR Reporter; however, it was flagged as a long event ( Fig. 5 ). Sanger sequencing further confirmed the long event as c.1175_1214del40 which was the expected variant according to CAP ( Fig. 6 ). One EQA DNA sample which did not have any variants was correctly identified as being negative for *BRCA* variants. Altogether, for a total of 31 DNA samples, covering a range of different variants including SNVs, deletions, and duplications in the *BRCA* genes, the analytical accuracy for variant detection was 93.55% (95% confidence interval [CI]: 78.58–99.21%). The analytical sensitivity and specificity were 92.86% (95% CI: 76.50–99.12%) and 100.00% (95% CI: 29.24–100.00%), respectively.

**Table 1:** Summary of variant detection by MASTR Reporter software for BRCA MASTR Plus assay (Multiplicom) on 10 cell line DNA from Coriell Institute and 21 DNA samples accrued from external quality assurance program

No.	DNA sample ID	Gene	Human Genome Variation Society (HGVS) nomenclature		MASTR Reporter		Zygosity	Concordance	Variant classification
			Coding DNA level	Protein level	Variant frequency (%)	VEP variant consequence/impact			
1	NA13714	*BRCA1*	c.5319dupC	p.Asn1774Glnfs*56	48.84	Frameshift/high	Heterozygous	Yes	Pathogenic
2	NA14091	*BRCA1*	c.5266dupC	p.Gln1756Profs*74	49.17	Frameshift/high	Heterozygous	Yes	Pathogenic
3	NA14624	*BRCA2*	c.5722_5723delCT	p.Leu1908Argfs*2	47.81	Frameshift/high	Heterozygous	Yes	Pathogenic
4	NA14639	*BRCA2*	c.6198_6199delTT	p.Ser2067Hisfs*10	53.73	Frameshift/high	Heterozygous	Yes	Pathogenic
5	NA14788	*BRCA2*	c.755_758delACAG	p.Asp252Valfs*24	50.00	Frameshift/high	Heterozygous	Yes	Pathogenic
6	NA14805	*BRCA2*	c.581G > A	p.Trp194*	49.30	Stop gained/high	Heterozygous	Yes	Pathogenic
7	NA14623	*BRCA2*	c.125A > G	p.Tyr42Cys	51.01	Missense/moderate	Heterozygous	Yes	Benign
8	NA14622	*BRCA2*	c.6275_6276delTT	p.Leu2092Profs*7	48.92	Frameshift/high	Heterozygous	Yes	Pathogenic
9	NA14170	*BRCA2*	c.5946delT	p.Ser1982Argfs*22	50.30	Frameshift/high	Heterozygous	Yes	Pathogenic
10	NA12878	*BRCA1 and BRCA2*	–	–	Refer to Fig. 3 and Fig. 4 for summaries	–	–	–	–
11	2016A01	*BRCA1*	c.5266dupC	p.Gln1756Profs*74	51.57	Frameshift/high	Heterozygous	Yes	Pathogenic
12	2016A02	*BRCA1*	c.4689C > G	p.Tyr1563*	49.53	Stop gained/high	Heterozygous	Yes	Pathogenic
13	2016A03	*BRCA2*	c.5946delT	p.Ser1982Argfs*22	50.22	Frameshift/high	Heterozygous	Yes	Pathogenic
14	2016B04	*BRCA1*	c.4327C > T	p.Arg1443*	51.01	Stop gained/high	Heterozygous	Yes	Pathogenic
15	2016B05	*BRCA1*	c.181T > G	p.Cys61Gly	52.79	Missense/moderate	Heterozygous	Yes	Pathogenic
16	2016B06	*BRCA1*	c.68_69delAG	p.Glu23Valfs*17	51.62	Frameshift/high	Heterozygous	Yes	Pathogenic
17	2017A01	*BRCA1*	c.1175_1214del40	p.Leu392Glnfs*5	>20	Identified as a long event ≥ 30nt	Heterozygous	No	Pathogenic
18	2017A02	*BRCA1*	c.2071delA	p.Arg691Aspfs*10	49.79	Frameshift/high	Heterozygous	Yes	Pathogenic
19	2017A03	*BRCA1 and BRCA2*	–	–	–	–	–	–	–
20	2017B04	*BRCA2*	c.581G > A	p.Trp194*	50.74	Stop gained/high	Heterozygous	Yes	Pathogenic
21	2017B05	*BRCA1*	c.3481_3491delGAAGATACTAG	p.Glu1161Phefs*3	53.20	Frameshift/high	Heterozygous	Yes	Pathogenic
		*BRCA2*	c.7630G > A	p.Gly2544Ser	50.54	Missense/moderate	Heterozygous	Yes	Uncertain significance
22	2017B06	*BRCA1*	c.1204delG	p.Glu402Serfs*8	51.62	Frameshift/high	Heterozygous	Yes	Pathogenic
23	2018A01	*BRCA1*	c.5138T > C	p.Val1713Ala	50.38	Missense/moderate	Heterozygous	Yes	Pathogenic
24	2018A02	*BRCA2*	c.6275_6276delTT	p.Leu2092Profs*7	51.90	Frameshift/high	Heterozygous	Yes	Pathogenic
25	2018A03	*BRCA1*	c.4327C > T	p.Arg1443*	48.76	Stop gained/high	Heterozygous	Yes	Pathogenic
26	2018B04	*BRCA1*	c.1175_1214del40	p.Leu392Glnfs*5	>20	Identified as a long event ≥ 30 nt	Heterozygous	No	Pathogenic
27	2018B05	*BRCA1*	c.181T > G	p.Cys61Gly	47.8	Missense/moderate	Heterozygous	Yes	Pathogenic
28	2018B06	*BRCA2*	c.581G > A	p.Trp194*	49.3	Stop gained/high	Heterozygous	Yes	Pathogenic
29	2019A01	*BRCA1*	c.4689C > G	p.Tyr1563*	51.6	Stop gained/high	Heterozygous	Yes	Pathogenic
30	2019A02	*BRCA2*	c.6275_6276delTT	p.Leu2092Profs*7	50.6	Frameshift/high	Heterozygous	Yes	Pathogenic
31	2019A03	*BRCA1*	c.4689C > G	p.Tyr1563*	48.9	Stop gained/high	Heterozygous	Yes	Pathogenic

## Discussion

Here we demonstrate the use of well-characterized cell line DNA and blinded proficiency testing samples for evaluation of a commercial MPS assay for the entire coding regions of the *BRCA1* and *BRCA2* genes in germline samples. In the absence of clinical samples, for a clinical laboratory starting a new assay, EQA samples and cell line samples are good resources for evaluating analytical accuracy and precision. Using these samples, we demonstrated the accurate identification of 10 different frameshift variants, 3 different stop gain variants, and 3 different SNVs in the *BRCA1* and *BRCA2* genes.

All samples showed concordance with the expected variants except for two EQA samples which harbor the same 40-bp deletion, c.1175_1214del40, in the *BRCA1* gene. First reported in 1994, 7 8 this deletion is not an uncommon pathogenic variant in HBOC patients (ClinVar, accessed on January 10, 2021). The inability to determine insertions and deletions spanning more than 30-bp is a declared limitation of the assay. Hence the laboratory may need to supplement the MPS assay with Sanger sequencing or use an alternative bioinformatics pipeline to analyze the sequencing data to confirm the exact deletion or insertion.

## Conclusion

In conclusion, we have shown high reproducibility and accuracy of the BRCA MASTR Plus assay on the MiSeq platform. The simple bench workflow in combination with rapid automated data analysis by the MASTR Reporter software make it suitable for use for germline *BRCA1* and *BRCA2* genetic testing in a clinical diagnostic laboratory. However, Sanger sequencing may still serve as a confirmatory method to improve diagnostic capability of the MPS assay.
